# 10th Anniversary of Cells—Advances in Plant, Algae and Fungi Cell Biology

**DOI:** 10.3390/cells11233759

**Published:** 2022-11-24

**Authors:** Suleyman I. Allakhverdiev

**Affiliations:** K.A. Timiryazev Institute of Plant Physiology, Russian Academy of Sciences, Botanicheskaya Street 35, Moscow 127276, Russia; suleyman.allakhverdiev@gmail.com

In 2021, the 10th anniversary of the publication of *Cells* occurred. To date, the journal has published more than 4500 papers, and the journal website attracts more than 60,000 monthly page views. To mark this important milestone, we organized a Special Issue entitled “10th Anniversary of Cells—Advances in Plant, Algae and Fungi Cell Biology”. For this Special Issue, we collected high-quality research articles in relevant research fields.

Cyanobacteria from the genus *Arthrospira/Limnospira* are considered haloalkalo-tolerant organisms with optimal growth temperatures of approximately 35 °C. They are most abundant in soda lakes in tropical and subtropical regions [1]. Indeed, the paper by Misztak et al. is devoted to the comprehensive genome-based characterization and physiological investigation of the new strain O9.13F, which was isolated from the winter freezing Solenoye Lake in Western Siberia [1]. Comparative genomics showed that no unique genes were found for the Siberian strain related to its tolerance to low temperatures and high salinity, while the fatty acid composition was specific and unique to this strain. The authors showed that the optimal cultivation temperature for strain O9.13F (20 °C) is lower than that for *Arthrospira/Limnospira* strains (35 °C) [1].

Coccoids are plant sap-sucking hemipterans that are considered serious pests in agriculture, horticulture, and forestry [2]. Szklarzewicz and coauthors [2] investigated the symbiotic systems of eight Polish species of scale insects of the Coccidae family: *Parthenolecanium corni*, *Parthenolecanium fletcheri*, *Parthenolecanium pomeranicum*, *Psilococcus ruber*, *Sphaerolecanium prunasti*, *Eriopeltis festucae*, *Lecanopsis formicarum* and *Eulecanium tiliae*. The histological, ultrastructural and molecular analyses showed that all these species host fungal symbionts in fat body cells. Analyses of ITS2 and beta-tubulin gene sequences, as well as fluorescence in situ hybridization, confirmed that they should all be classified into the genus *Ophiocordyceps*. *Ophiocordyceps* fungi are commonly known as virulent, specialized entomopathogens. The essential role of the fungal symbionts observed in the biology of the soft scale insects examined was confirmed by their transovarial transmission between generations [2].

Another thing to note is that the arbuscular mycorrhizal fungus *Glomus Viscosum* proves tolerant to *Verticillium* wilt in artichoke by modulating antioxidant defense systems [3]. The artichoke (*Cynara scolymus* L.) is a horticultural species of relevant economic interest belonging to the Asteraceae family that is widely cultivated in the Mediterranean basin and widespread throughout the world [3]. Several studies have demonstrated that even some nonfood byproducts of artichokes are widely used as hepatoprotective, antioxidant, anticarcinogenic, hypoglycemic, and hypocholesterolemic agents (see [3]). The health-promoting properties and important nutritional values of artichokes have been extensively related to the high content of some bioactive phenolic compounds, such as caffeoylquinic acid derivatives and flavonoids, showing strong scavenging activity against reactive oxygen species (ROS) and free radicals [3]. On the other hand, Verticillium wilt, caused by the fungal pathogen *Verticillium dahliae*, is the most severe disease threatening artichoke (*Cynara scolymus* L.) plants. Villani et al. evaluated the effect of the AMF *Glomus viscosum Nicolson* in enhancing plant tolerance towards the pathogen *V. dahliae*. The role of the antioxidant systems involved in the complex network of the pathogen–fungi–plant interaction was investigated. The results obtained showed that the AMF-inoculated plants exhibited significant increases in the activities of antioxidant enzymes, a higher content of ascorbate (ASC) and glutathione (GSH), and a decrease in the levels of lipid peroxidation and hydrogen peroxide (H_2_O_2_). Hence, *G. viscosum* may represent an effective strategy for mitigating *V. dahliae* pathogenicity in artichokes, improving nutritional value and benefiting human health [3].

The chlorococcal green alga *Parachlorella kessleri* is a favorable organism, as it can produce both starch and neutral lipids, and *P. kessleri* commonly divides into more than two daughter cells by means of a specific mechanism—multiple fission. Zachleder et al. used synchronized cultures of algae to study the effects of supra-optimal temperatures [4]. Synchronized cultures were grown at optimal (30 °C) and supra-optimal (40 °C) temperatures and incident light intensities of 110 and 500 µmol photons m^−2^s^−1^. The time course of cell reproduction (*DNA* replication, cellular division), growth (total *RNA*, protein, cell dry matter, cell size), and synthesis of energy reserves (net starch, neutral lipid) was studied [4]. At 40 °C, cell reproduction was arrested, but the growth and accumulation of energy reserves continued; this led to the production of giant cells enriched in protein, starch, and neutral lipids [4].

The plant hormone cytokinin (CK) is central to plant life, regulating many processes. Pizarro et al. investigated the effects of CK on cellular trafficking and on the cytoskeleton in plant cells [5]. They found that in addition to the xylanase receptor-like protein (RLP) LeEIX2, CK affects the distribution of the flagellin receptor-like kinase (RLK) flagellin-sensing 2 (FLS2), increasing its presence in the plasma membrane. FLS2, first characterized in Arabidopsis, acts as the PRR for the bacterial PAMP (pathogen-associated molecular pattern) flagellin in several plant species (see [5]). Examining cellular trafficking compartments and the cytoskeleton in general, the authors showed that CK affects endosome distribution and increases the number of endomembrane compartments. CK also caused disorganization and reduction in actin filaments, but not tubulin. The results are in agreement with those previously reported for fungi, suggesting a fundamental role for CK in regulating cellular integrity and trafficking as a mechanism for controlling and executing CK-mediated processes [5].

The identification of putative virulence genes by means of DNA methylation studies in the cereal pathogen *Fusarium graminearum* was described by Tini et al. [6]. The *DNA* isolated from SC50 and SC50 × 3 was subjected to a methylation content-sensitive enzyme and a double-digest, restriction-site-associated *DNA* technique (ddRAD-MCSeEd). *DNA* methylation analysis indicated 1024 genes whose methylation levels changed in response to inoculation on a healthy host after subculturing. These results demonstrate that the physiological shifts following subculturing have an impact on genomic *DNA* methylation levels and suggest that the ddRAD-MCSeEd approach can be an important tool for detecting genes potentially related to fungal virulence [6].

Ivanov et al. examined the potential for pilot-scale starch production in *C. reinhardtii* using a supraoptimal temperature, a method that has already been proven to cause a rapid 2-fold increase in starch yields under laboratory conditions [7]. The experiments described in this paper are the first attempt to employ a supraoptimal temperature in the production of starch in microalgae at the pilot scale and showed that exposure to a supraoptimal temperature (39 °C) causes a complete block of nuclear and cellular division accompanied by an increased accumulation of starch. The method was successfully applied and resulted in an almost 3-fold increase in the starch content of *C. reinhardtii* dry matter. Nevertheless, technical challenges, such as bioreactor design and light availability within the culture, still need to be addressed (see [7]).

It has been challenging to simultaneously improve photosynthesis and stress tolerance in plants. Liu et al. hypothesized that the ectopic expression of a CAM-specific phosphoenolpyruvate carboxylase (PEPC) would enhance both photosynthesis and abiotic stress tolerance [8]. Their experiments showed a higher photosynthetic rate and biomass production under normal conditions, along with significant carbon metabolism changes in malate accumulation, the carbon isotope ratio δ13C, and the expression of multiple orthologues of CAM-related genes. Furthermore, AaPEPC1 overexpression enhanced proline biosynthesis and improved salt and drought tolerance in the transgenic plants [8].

Zinc is implicated in numerous cellular processes, including cell division and DNA and protein synthesis, as well as in the catalytic activity of many enzymes. Two major membrane protein families facilitate zinc homeostasis in the animal kingdom, i.e., Zrt/Irt-like proteins and Zn transporters (ZnTs), which essentially conduct zinc flux in opposite directions. Human ZIPs (hZIPs) regulate the importing of extracellular zinc to the cytosol, being critical in preventing the overaccumulation of this potentially toxic metal and crucial for diverse physiological and pathological processes, including the development of neurodegenerative disorders and several cancers [9]. Becares et al. reported that yeast is able to produce four full-length hZIP members belonging to three different subfamilies [9]. One target (hZIP1) was purified in the high quantity and homogeneity required for downstream biochemical analysis.

The primary purpose of the review by Barre et al. is to give an overview of the types of glycans present in the glycan shield of different pathogenic enveloped viruses and how legume lectins with different specificities can act as carbohydrate-binding agents (CBAs) for these viruses. In addition, the biomedical and therapeutic potential of plant lectins as antiviral drugs has been discussed [10].

Plants regularly encounter a wide range of abiotic and biotic stresses in nature. Abiotic stress includes drought, salinity, extreme temperatures, radiation, floods, and heavy metals, whereas biotic stressors include insects, animal herbivores, and microbial pathogens [11]. Plants have evolved various strategies to detect herbivores and mount an effective defense system against them. Interestingly, ion channels or transporters are the first responders against herbivores. A review by Gandhi et al. described a comprehensive overview of the role of ion channels in early defense signaling against herbivorous insects [11].

A review by Hameed et al. was dedicated to the effects of salt stress on chloroplasts, their structures, and various biochemical reactions occurring in them [12]. The authors showed that the presence of salt in plant cells disrupts basic metabolic processes, contributing to severe negative effects on plant development and growth. For example, changes in chloroplast size, number, lamellar organization, lipid and starch accumulation interfere with cross-membrane transportation. Research has shown that the maintenance of normal chloroplast physiology is necessary for the survival of the entire plant [12].

The core abscisic acid (ABA) signaling pathway consists of receptors, phosphatases, kinases and transcription factors, among them ABA INSENSITIVE 5 (ABI5) and ABRE BINDING FACTORs/ABRE-BINDING PROTEINs (ABFs/AREBs), which belong to the BASIC LEUCINE ZIPPER (bZIP) family and control the expression of stress-responsive genes. ABI5 is mostly active in seeds and prevents germination and postgerminative growth under unfavorable conditions. The review by Collin et al. focuses on recent reports regarding ABI5 and ABF/AREB functions during abiotic stress [13]. They discuss the increased stress tolerance of transgenic plants overexpressing genes encoding ABA-dependent bZIPs. The authors suggest that ABI5 and ABFs/AREBs are crucial ABA-dependent transcription factors regulating processes essential for plant adaptation to stress at different developmental stages [13].

In the review paper by Sharkey [14], the pentose phosphate pathway (PPP) is divided into an oxidative branch that makes pentose phosphates and a nonoxidative branch that consumes pentose phosphates, although the nonoxidative branch is considered reversible. The reaction sequence in the Calvin–Benson cycle is the opposite of the typical direction of the nonoxidative PPP. This can be considered an anabolic version of the nonoxidative PPP. In addition to the strong association between the nonoxidative PPP and photosynthesis metabolism, there is recent evidence that oxidative PPP reactions form a shunt around the nonoxidative PPP section of the Calvin–Benson cycle. A constitutive operation of this shunt occurs in the cytosol and gives rise to an unusual labelling pattern of photosynthetic metabolites, while an inducible shunt in the stroma may occur in response to stress (see [14]).

We would like to express our sincerest thanks to our readers, innumerable authors, anonymous peer reviewers, editors, and all the people working in some way for the journal who have made substantial contributions for years. Without your support, we would never have made it.

For the details of the *Cells* 2021 Best Paper Awards for Anniversary Special Issues please click here https://www.mdpi.com/journal/cells/awards.pdf/0/pdf_32_2021_2_award.pdf (accessed on 28 February 2022).

## Data Availability

The datasets generated during and/or analyzed during the current study are available from the corresponding author on reasonable request.
